# Role of IRAK-M in Alcohol Induced Liver Injury

**DOI:** 10.1371/journal.pone.0057085

**Published:** 2013-02-21

**Authors:** Yipeng Wang, Youjia Hu, Chen Chao, Muhammed Yuksel, Isabelle Colle, Richard A. Flavell, Yun Ma, Huiping Yan, Li Wen

**Affiliations:** 1 The Sections of Endocrinology, Yale University School of Medicine, New Haven, Connecticut, United States of America; 2 Department of Immunobiology, Yale University School of Medicine, New Haven, Connecticut, United States of America; 3 Clinical Research Centre for Autoimmune Liver Disease, Beijing You-an Hospital, Capital Medical University, Beijing, China; 4 Department of Gastroenterology, Ghent University Hospital, Belgium; 5 Institute of Liver Study, King’s College School of Medicine, University of London, London, United Kingdom; Charité, Campus Benjamin Franklin, Germany

## Abstract

Increasing evidence suggests that innate immunity plays an important role in alcohol-induced liver injury and most studies have focused on positive regulation of innate immunity. The main objective of this study was to investigate the negative regulator of innate immunity, IL-1/Toll-like receptor (TLR) signaling pathways and interleukin receptor-associated kinase-M (IRAK-M) in alcoholic liver injury. We established an alcohol-induced liver injury model using wild type and IRAK-M deficient B6 mice and investigated the possible mechanisms. We found that in the absence of IRAK-M, liver damage by alcohol was worse with higher alanine transaminase (ALT), more immune cell infiltration and increased numbers of IFNγ producing cells. We also found enhanced phagocytic activity in CD68^+^ cells. Moreover, our results revealed altered gut bacteria after alcohol consumption and this was more striking in the absence of IRAK-M. Our study provides evidence that IRAK-M plays an important role in alcohol-induced liver injury and IRAK-M negatively regulates the innate and possibly the adaptive immune response in the liver reacting to acute insult by alcohol. In the absence of IRAK-M, the hosts developed worse liver injury, enhanced gut permeability and altered gut microbiota.

## Introduction

Alcoholic liver disease (ALD) comprises a spectrum of liver disorders that include alcoholic fatty liver, alcoholic steatohepatitis, liver cirrhosis and possibly hepatic carcinoma [1], [2], [3], [4]. The pathogenesis of ALD is multi-factorial and affects different cell types in the liver with diverse consequences [5], [6]. In addition to the direct effects of ethanol and its toxic metabolites on different cell types in the liver, increasing evidence suggests that innate immunity plays an important role in the pathogenesis of ALD [2], [7], [8]. IL-1/Toll-like receptor (TLR) signaling pathways and interleukin receptor-associated kinase (IRAK) family are critical molecules in innate immunity playing important roles in anti-pathogen responses, inflammation and autoimmunity [9], [10]. IRAK-M is a member of IRAK family that is mainly expressed on macrophages and mononuclear cells [11], [12]. IRAK-M negatively regulates TLR signaling through inhibition of MyD88, the adaptor molecule downstream of most of the TLRs [11], [12], [13]. Macrophages from IRAK-M deficient mice showed enhanced NF-κB activity and elevated expression of inflammatory cytokines upon stimulation with several TLR ligands [11], [14], [15]. Moreover, IRAK-M−/− mice had increased inflammatory responses to bacterial infection [11].

Kupffer cells, the liver resident macrophages, usually express CD68, contribute to the most of the effects of alcohol-associated liver damage including alcohol-induced oxidative stress on the hepatocytes [16], [17] and enhanced inflammatory cytokine production [18], [19]. Furthermore, alcohol exposure increased gut permeability, which led to an elevated level of intestinal endotoxin (LPS) in the liver and circulation [20], [21]. It is known that TLR4 is the receptor for LPS [22], [23] and therefore, it is not surprising that TLR4 plays an important role in ALD [24], [25], [26], [27].

In the current study, we intended to establish a murine model of acute alcoholic liver damage using wild type and IRAK-M deficient B6 mice in order to investigate the role of IRAK-M, the negative regulator of innate immune system, in alcohol-induced liver damage. We have found that IRAK-M deficient mice appeared to be more susceptible to alcohol-induced liver damage that was accompanied by enhanced inflammation, gut permeability and altered intestinal microbiota.

## Materials and Methods

### Animals and Reagents

Wild type C57BL/6 mice were obtained originally from the Jackson Laboratories (Bar Harbor, Maine) and maintained at the Yale Animal Facility. IRAK-M−/− mice were generated as described previously [11] and back-crossed on C57BL/6 background for 10 generations (http://jaxmice.jax.org/strain/007016.html). The genetic purity of the IRAK-M−/− mice was further confirmed by mouse genome SNP analysis using 1449 Illumina beadchip (www.dartmouse.org). All the mice used in this study were 6 to 8 weeks old and housed in specific pathogen–free conditions with autoclaved food and bedding in individually ventilated filter cages. All studies were approved by the Institutional Animal Care and Use Committee of Yale University.

All the monoclonal antibodies used in this study were purchased from BioLegend (San Diego, CA) or eBioscience (San Diego, CA).

### Alcohol Treatment

Mice were treated with 10% ethanol in drinking water for 7 days and gavaged with 200 µl 60% ethanol (6 g/kg) on day 7. Mice were sacrificed 16 hours after gavage. The control mice were given normal water and gavaged with 200 µl water on day 7.

### Isolation of Liver Mononuclear Cells

Mouse liver was first perfused with PBS via the portal vein and the liver tissue was then ground between two single frosted slides to obtain a single cell suspension. Liver mononuclear cells (LMNCs) were harvested at the interface of a 40% and 80% Percoll gradient (GE Healthcare, Piscataway, NJ) after discontinuous gradient centrifugation. Residual red blood cells (RBC) were lysed with RBC lysis buffer (eBiosience, San Diego, CA). The LMNCs were then washed twice with PBS.

### Phagocytosis Assay

FITC-dextran (mw 40,000, Sigma) was used as a phagocytic tracker in the phagocytosis assay. LMNCs were incubated with 1 mg/ml FITC-dextran at 37°C (binding and uptake) or 4°C (non-specific binding) for 1 hr in culture medium. After washing, the cells were stained with different fluorochrome-conjugated mAbs and phagocytic capacity of LMNCs was examined by FITC-dextran uptake using flow cytometry.

### Flow Cytometry Analysis

The expression of different surface markers and intracellular cytokines (ICC) in LMNCs was analyzed by flow cytometry. LMNCs were first incubated with Fc block (2G4, eBioscience) at 4°C for 15 min followed by further incubation with different fluorochrome-conjugated mAbs at optimal concentrations for 30 min. The cells were then washed twice with PBS containing 2% FBS and fixed in 1% paraformaldehyde prior to data collection by flow cytometry. For ICC staining, LMNCs were stimulated with PMA (20 ng/mL), Ionomycin (1 µmol/L) and Golgi plug (1%) for 5 hrs. Cell surface markers and ICC were then stained after stimulation according to the manufacturer’s protocol (eBioscience). Data were collected using a LSRII flow cytometer (BD Biosciences) and data analysis was performed using FlowJo software (TreeStar).

### Serum Alanine Aminotransferase (ALT) Activity

The level of serum ALT was measured using a commercial kit (Cayman Chemical, Ann Arbor, MI) according to the manufacturer’s instructions.

### Intestinal Permeability Assay

To determine intestinal permeability, mice were gavaged with FITC-dextran (Sigma) in PBS at 6 mg/g body weight. Peripheral blood samples were collected 4 hrs after gavage. After 1∶2 dilution, circulating FITC-dextran was measured by fluorescence spectrophotometry (PerkinElmer 1420 Multilabel Counter plate reader) at the excitation wavelength of 485 nm and emission wave length of 535 nm. The concentration of FITC-dextran was calculated according to the standard curve in which diluted normal mouse serum was mixed with known concentrations of FITC-dextran.

### Endotoxin LAL Assay

LPS level in serum was tested using chromogenic endotoxin LAL assay kit (GeneScript) according to the manufacturer’s instructions.

### Analysis of Culturable Bacteria in Mouse Feces

Fresh feces from the mice used in this study were collected and weighed. The same amount of feces was resuspended in 500 µl sterile PBS and vortexed thoroughly. 100 µl from each diluted sample was spread on LB and blood agar plates followed by incubation at 37°C in for 24 hrs. For culturable anaerobic bacteria, the plates were placed in GasPak EZ Anaerobe Container System (BD, Sparks, MD) and incubated for 48 hrs. Bacterial colonies were counted from each plate. This assay was performed in duplicate for each sample and the data are presented as the number of culturable bacterial colonies per mg feces.

### Ratios of Gram-positive (G^+^) and Gram-negative (G^−^) Bacteria in Feces

Bacterial DNA of mouse feces was isolated as previously described [28] with some modification. Briefly, the collected feces sample was resuspended in 300 µl TE and then 5 freeze-thaw cycles were carried out. 70 µl lysozyme (200 µg/ml, Sigma) was added followed by a 3-hour incubation at 37°C. 20 µl SDS (10%) and 2 µl Proteinase K (20 mg/ml) were then added and the samples incubated for an additional hour at 37°C. The samples were further incubated for 20 minutes at 65°C after adding saturated (5M) NaCl (72 µl) and 0.1 g glass beads (0.1 mm, Biospec Inc). DNA was extracted using phenol:chloroform:isoamyl alcohol (25∶24∶1) and precipitated with 100% ethanol.

The DNA content of G^+^ and G^−^ bacteria was quantified by qPCR (IQ5, Bio-Rad, Hercules, CA) using G^+^ and G^−^ specific forward premiers (G^+^-F and G^−^-F) and reversal primer for universal bacterial. Total 16S DNA was used as positive control and the result was analyzed by delta-delta CT method after normalization with 16S DNA. G^+^/G^−^ ratio was calculated. Each sample was analyzed in triplicate and the experiments were repeated twice. All the samples were negative for 18S DNA indicating that there was no eukaryotic cell contamination.

### Statistical Analysis

Statistical analysis was performed using GraphPad Prism software. Non-parametric two-way ANOVA was used in most experiments and *P* values of less than 0.05 were considered significant.

## Results

### Mouse Genome Analysis

**Figure 1:**
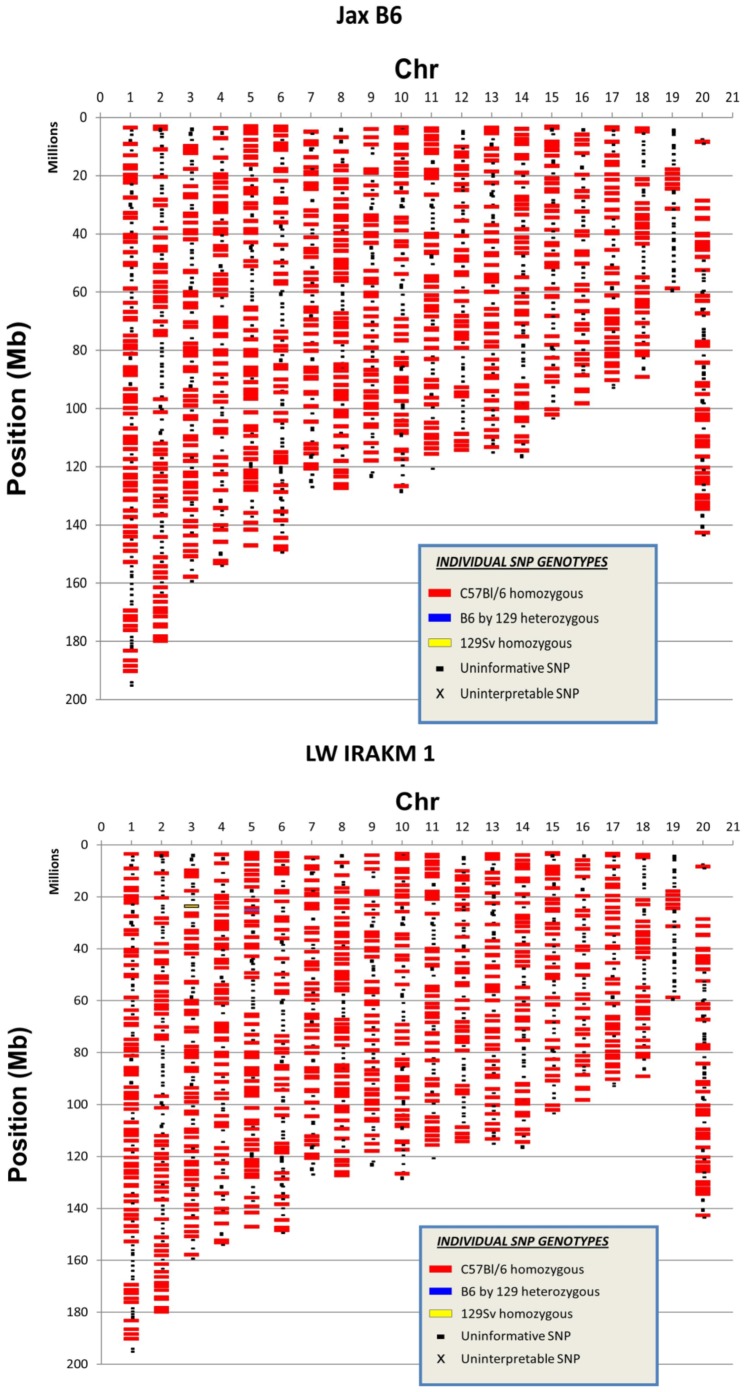
Genome analysis. The genetic purity of IRAK-M−/− B6 mice was analyzed with genomic DNA from IRAK-M−/− B6 mice (breeders). WT B6 mice from the Jackson Laboratory were used as controls. Genomic SNP analysis of one WT control (upper) and one IRAK-M−/− mouse (lower) is shown in the figure.

One pitfall of using genetically engineered mice is the purity of the mouse strain, as genomic contamination could affect the data interpretation. The genetic purity of the IRAK-M−/− mice used in this study was analyzed by mouse genome SNP analysis (www.dartmouse.org). The genomic DNA IRAK-M−/− mice were tested by SNP using Illumina bead chip. We used C57B/L6 mice from the Jackson Laboratory as controls, and these mice were used to backcross the IRAK-M−/− mice. SNP test showed that our IRAK-M−/− mice were fully back-crossed to B6 genetic background (Figure 1).

### Alcohol Induced Worse Liver Damage in IRAK-M−/− Mice

**Figure 2:**
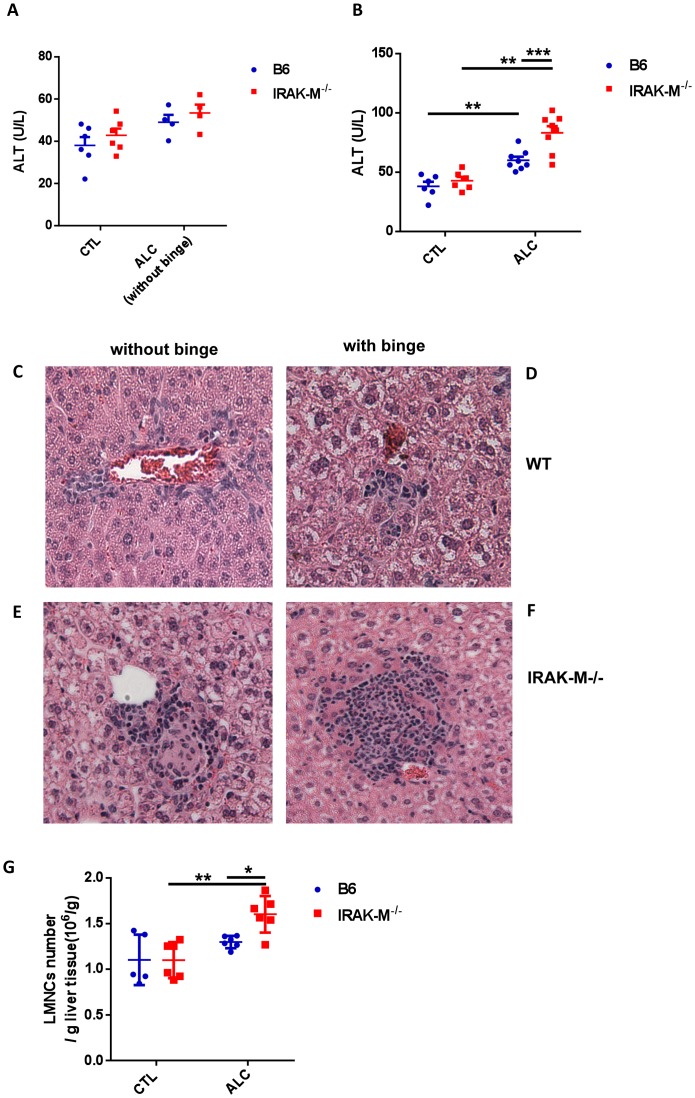
Liver injury after alcohol treatment in IRAK-M−/− mice. To induce alcoholic liver damage, mice were fed with alcohol as described in *Materials & Methods*. (A) Serum ALT levels in control (CTL) and 10% alcohol treated (ALC, without binge) IRAK-M−/− mice (red) compared with wild type B6 mice (blue). Data shown are from one of 4 experiments. (B) Serum ALT levels in control (CTL) and alcohol (10% and binge) treated (ALC) IRAK-M−/− mice (red) compared with wild type B6 mice (blue). Data are from pooled 2 experiments. (C-F): Liver tissue was stained with standard H&E and liver histology was viewed under a light microscope (20X) by a blinded investigator. Data represent one of two independent experiments (n = 3–4 per group in each experiment). (C+D) Liver histology from a WT B6 mouse without (C) and with binge ALC exposure (D). (E+F) Liver histology from an IRAK-M−/− B6 mouse without (E) and with binge ALC exposure (F). (G) Absolute number of LMNCs per gram liver tissue in control and alcohol treated mice. More infiltrating lymphocytes were found in IRAK-M−/− mice (red) compared with wild type B6 mice (blue) after binge alcohol treatment. Data were from 2 pooled experiments and error bars represent the *SD* of samples within a group. The experiment was repeated twice. *P<0.05, **P<0.01, Two way ANOVA analysis.

To study the role of innate immunity, in particular IRAK-M, in alcohol-induced liver damage, we treated wild type (WT) and IRAK-M−/− B6 mice with alcohol as described in the Materials and Methods. 10% alcohol in drinking water was administered to mimic a daily light alcohol consumption and the single gavage with a larger amount of alcohol (60% alcohol in 200 µl, ∼6 g/kg) was to mimic an alcoholic binge, which has been reported to be one of the main triggers of alcoholic liver damage in human [29]. There was very mild liver damage induced by daily 10% alcohol water consumption in both WT and IRAK-M−/− mice, indicated by serum ALT levels (Figure 2A) and liver histology (Figure 2C and 2E, without binge). However, the difference between WT and IRAK-M−/− was negligible (Figure 2A) although it appeared that IRAK-M−/− mice showed more liver damage (Figure 2E). In contrast, a single episode of heavy alcohol consumption triggered liver inflammation and injury as evidenced by increased serum ALT levels in both WT and IRAK-M−/− mice (Figure 2B) and LMNC infiltration in the liver of IRAK-M−/− mice (Figure 2D and 2F). We also examined the absolute number of LMNC infiltration per gram liver tissue analyzed, and the results were consistent (Figure 2G). We found that the absolute number of LMNCs was higher in IRAK-M−/− mice than in WT B6 mice after binge alcohol consumption (Figure 2G), suggesting that IRAK-M acts as a negative regulator for alcohol-induced steatohepatitis.

### Increased Number of T Cells and CD68^+^ Cells and Decreased Foxp3^+^ Treg Cells in the Liver of IRAK-M−/− Mice after Alcohol Treatment

**Figure 3:**
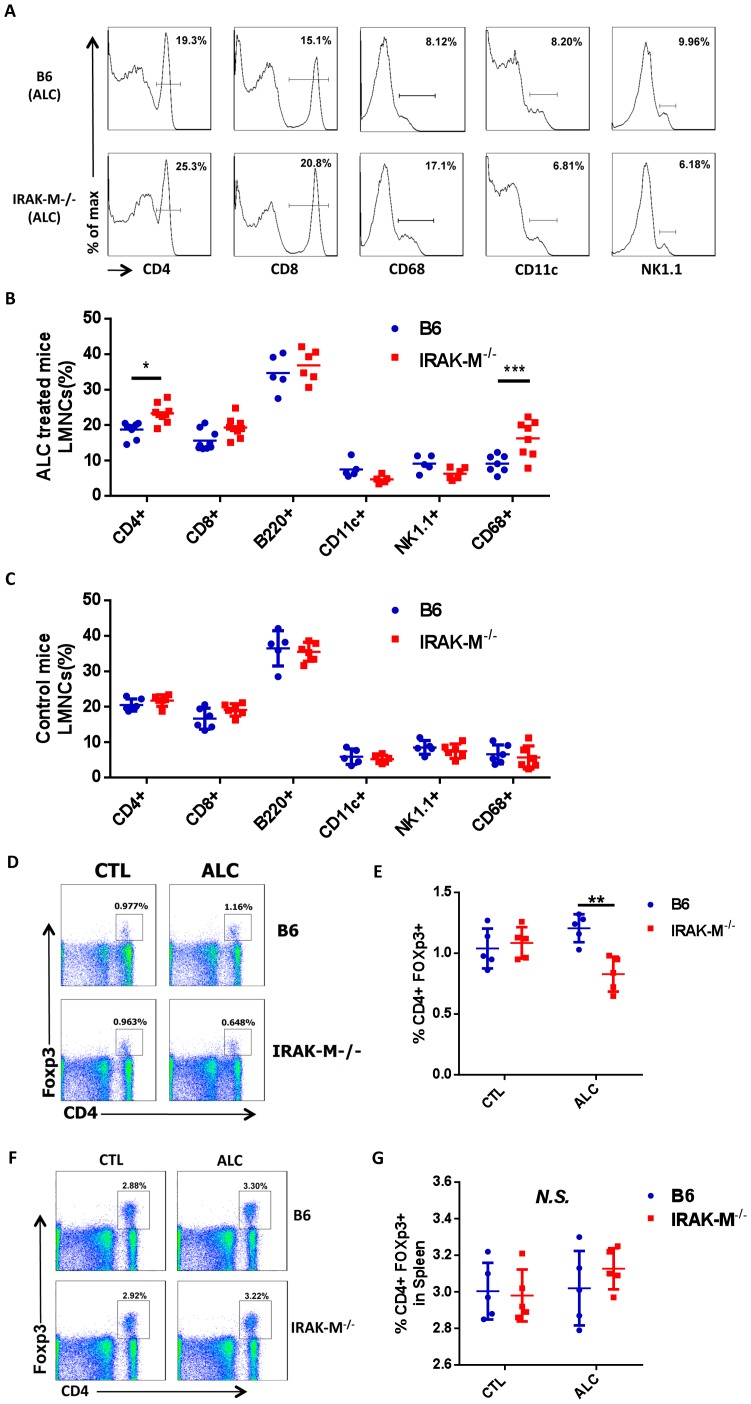
Flow cytometric analysis of immune cell composition in liver after alcohol treatment. (A) Representative histograms of liver mononuclear cells (LMNCs) in wild type B6 mice (upper panel) and IRAK-M−/− mice (lower panel). (B) Summary of immune cell composition of LMNC in wild type B6 (blue) and IRAK-M−/− mice (red) in ALC groups. (C) Summary of immune cell composition of LMNC in wild type B6 (blue) and IRAK-M−/− mice (red) in CTL groups. (D) Representative dot plots of Treg (CD4^+^Foxp3^+^) cells in total LMNCs of control (CTL, left panel) and binge alcohol treated (ALC, right panel) WT B6 (upper panel) and IRAK-M−/− mice (lower panel). (E) Summary of percentage of Treg cells in total LMNCs of CTL and ALC treated WT B6 (blue) and IRAK-M−/− B6 (red) mice. (F) Representative dot plots of Treg (CD4^+^Foxp3^+^) cells in total splenocytes of control (CTL, left panel) and alcohol treated (ALC, right panel) WT B6 (upper panel) and IRAK-M−/− mice (lower panel). (G) Summary of percentage of Treg cells in total splenocytes of CTL and ALC treated WT B6 (blue) and IRAK-M−/− B6 (red) mice. Error bars represent the *SD* of samples within a group. The experiment was performed 3 times and the data presented are from one of the 3 experiments. **P<0.01, Two way ANOVA analysis. *N.S.* not statistically significant.

To identify the cell type infiltrating liver tissue, we extracted the infiltrated LMNCs from livers of IRAK-M−/− and WT B6 mice and analyzed by flow cytometry after staining with a panel of immune cell markers. As shown in Figure 3A–B, more CD4^+^ T cells were found in the livers of IRAK-M−/− mice than WT B6 mice after alcohol treatment. CD68^+^ cells [30] were also significantly increased among the infiltrated LMNCs in IRAK-M−/− mice (Figure 3A–B).

There was no difference in B cells (B220^+^CD19^+^) (Fig. 3B) and CD11b^+^ macrophages (data not shown) in LMNCs from IRAK-M−/− and WT B6 mice after alcohol exposure. As expected that there was no difference in LMNCs from control PBS treated WT or IRAK-M−/− mice (Figure 3C). To study whether binge alcohol consumption would affect Treg cells in the liver, we examined CD4^+^Foxp3^+^ Treg cells in LMNCs. Despite an increase in CD4^+^ T cells in LMNCs from IRAK-M−/− mice, we found a significant decrease of CD4^+^Foxp3^+^ Treg cells in the liver of alcohol treated IRAK-M−/− mice compared to WT B6 mice (Figure 3D and 3E). The decrease of CD4^+^Foxp3^+^ Treg cells appeared to be restricted to liver, as we did not find any obvious changes in other lymphoid tissues including spleen (Figure 3F and 3G). There was also no difference in Treg cells in non-alcohol treated IRAK-M−/− and WT B6 mice (Figure 3D–G).

### Increase in IFNγ Producing CD8 T Cells in Liver of IRAK-M−/− Mice after Alcohol Treatment

**Figure 4:**
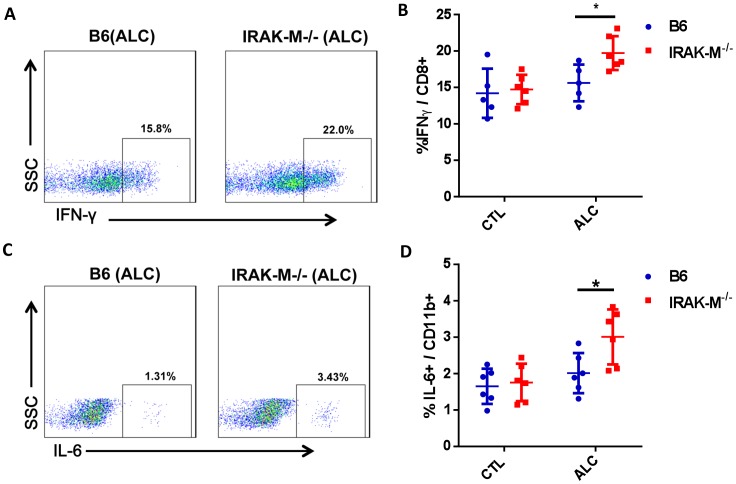
Inflammatory cytokine in LMNCs. *Ex vivo* LMNCs were stained with intracellular cytokines and different surface markers as described in *Materials and Methods.* (A) Representative FACS plots showing IFNγ^+^ cells after gating CD8^+^ T cells in alcohol treated mice. (B) Summary of percentage of IFNγ producing CD8 T cells in LMNCs of control (CTL) and alcohol treated (ALC) B6 (blue) and IRAK-M−/− mice (red). (C) Representative FACS plots showing IL-6 producing (CD11b^+^Kupffer cells after gating CD11b^+^ LMNCs in alcohol treated mice. (D) Summary of percentage of IL-6 producing CD11b^+^ Kupffer cells in LMNCs of control (CTL) and alcohol treated (ALC) B6 (blue) and IRAK-M−/− mice (red). Error bars represent the *SD* of samples within a group. Experiments were performed 4 times and n = 2−4 in each group of each experiment. The data presented are from two pooled experiments. *P<0.05, (Two-way ANOVA test).

To determine if there were any functional changes in LMNCs in response to alcohol consumption, we examined inflammatory cytokine producing cells in LMNCs. We found that there was a significant increase in IFNγ producing CD8^+^ T cells in LMNCs of IRAK-M−/− mice after alcohol consumption compared to WT B6 mice (Figure 4A and 4B). There was also a significant increase in pro-inflammatory cytokine IL-6 production by CD11b^+^ cells (regardless of the expression of CD68) in LMNCs of IRAK-M−/− mice compared with WT B6 mice (Figure 4C and 4D). It is interesting that despite the increase of CD4^+^ T cells in LMNCs of IRAK-M−/− mice after alcohol consumption, we did not find any obvious difference in pro-inflammatory cytokine production by these cells comparing the IRAK-M deficient and sufficient mice (data not shown). We also investigated other pro-inflammatory (TNFalpha, IL-12, IL-17) and anti-inflammatory (IL-4, IL-10) cytokines in LMNCs and did not find significant changes in any subset of LMNCs comparing IRAK-M−/− and WT B6 mice.

### Increased Phagocytic Function of LMNC and CD68^+^ Cells in Liver of IRAK-M−/− Mice after Alcohol Treatment

**Figure 5:**
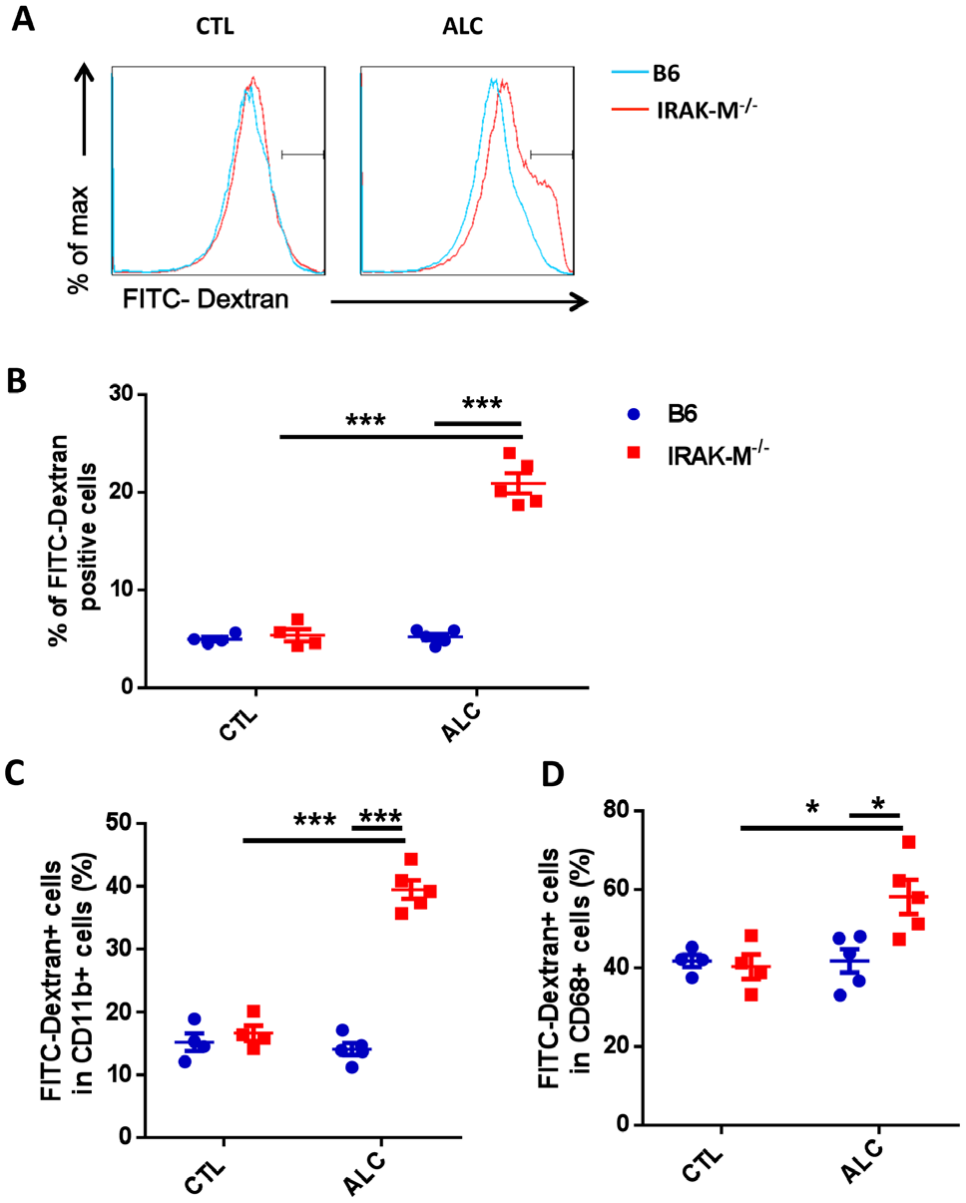
Phagocytic activity of Kupffer cells in liver after alcohol treatment. (A) Representative histogram of FITC-dextran intake LMNCs in wild type B6 mice (blue line) and IRAK-M−/− mice (red line, 2%). (B) FITC-dextran uptake by LMNCs in wild type B6 (blue) and IRAK-M−/− mice (red, 23%). (C) FITC-dextran uptake by CD11b^+^ Kupffer cells in wild type B6 (blue) and IRAK-M−/− mice (red). (D) FITC-dextran uptake by CD68^+^ Kupffer cells in wild type B6 (blue) and IRAK-M−/− mice (red). Experiments were performed 3 times. N = 3–4 in each group of each experiment. The data presented are from one of the 3 experiments. Error bars represent the *SD* of samples within a group. *P<0.05, **P<0.01, ***P<0.001, Two way ANOVA test.

We showed earlier that binge alcohol consumption induced a significant increase in CD68^+^ cells in IRAK-M−/− mice. To study whether alcohol consumption would also alter the function of CD68^+^ cells, we tested phagocytic activity using LMNCs from IRAK-M−/− and WT B6 mice after alcohol treatment as described in *Materials and Methods*. LMNCs from IRAK-M−/− mice showed increased phagocytic activity, as more FITC-Dextran intake was observed in LMNCs from IRAK-M−/− mice than from WT B6 mice (Figure 5A–B). Further analysis revealed that there was a ∼4-fold increase in CD11b^+^/FITC-Dextran positive cells in LNMCs of IRAK-M−/− mice compared with B6 mice (Figure 5C). Similarly, CD68^+^ cells from IRAK-M−/− mice also expressed higher phagocytic function (more FITC-Dextran intake) than in B6 mice (Figure 5D). CD11b^+^ Kupffer cells have been characterized as cytokine-producing cells [30], which supported our data presented earlier showing more IL-6-producing CD11b^+^ cells in LMNCs from IRAK-M−/− mice (Figure 4D).

### Intestinal Permeability is Up-regulated after Acute Alcohol Treatment in IRAK-M−/− Mice

**Figure 6:**
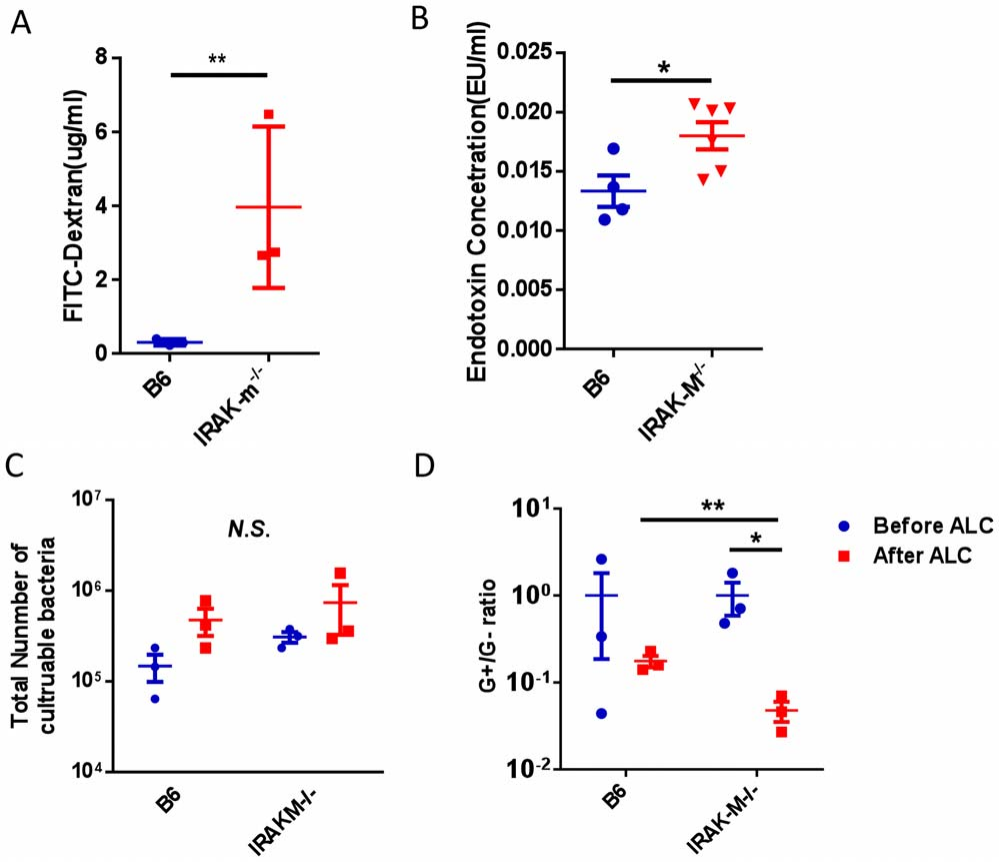
Altered gut permeability and composition of gut bacteria in the intestine after alcohol treatment. (A) FITC-dextran concentration in blood after gut permeability test in wild type B6 mice (blue) and IRAK-M−/− mice (red). (B) LPS content in the blood of B6 mice (open bar) and IRAK-M−/− mice (solid bar). (C) Number of culturable bacteria in the intestine before (blue) and after (red) binge alcohol treatment (ALC) in wild type B6 and IRAK-M−/− mice. (D) G^+^/G^−^ gut bacteria ratio from mouse feces tested by Q-PCR before (blue) and after (red) binge alcohol treatment in wild type B6 and IRAK-M−/− mice. Experiments were performed 3 times for A and twice for B, C and D. The data presented in A, C and D were from one of the experiments, and those shown in B were from pooled 2 experiments. n = 2–3 in each group of each experiment. Error bars represent the *SD* of samples within a group. *P<0.05, **P<0.01 (Student’s t-test).

It has been reported that the liver-gut axis plays an important role in the progression of ALD [24]. To test whether binge alcohol consumption would change gut permeability in our model system, we studied gut permeability after a single alcohol treatment in IRAK-M−/− and WT B6 mice, as described in *Materials and Methods*. It was interesting that short term alcohol consumption led to a marked increase in intestine leakiness measured by FITC-Dextran in the circulation in IRAK-M−/− mice whereas intestine permeability in WT B6 mice did not show significant change after the short term alcohol consumption (Figure 6A). To test whether increased gut permeability would lead to the leakiness for LPS, a major Gram-negative bacterial product in the intestine, we then tested LPS content in the circulation. As shown in Figure 6B, serum level of LPS from IRAK-M−/− mice was much higher than from WT B6 mice and the results supported the notion that a “leaky” gut was associated with the increased level of LPS in circulation.

### Altered Gut Bacteria after Acute Alcohol Treatment

To further investigate whether alcohol consumption would alter gut flora and lead to an overgrowth of Gram-negative bacteria in the intestine, which might be another factor contributing to the increased level of LPS, we collected mouse feces and analyzed total culturable bacteria. More bacteria were found in both WT B6 and IRAK-M−/− mice after alcohol consumption although this was not statistically significant (Figure 6C). We further investigated the Gram-negative (G^−^) and Gram-positive (G^+^) bacterial content in the feces by real time PCR. In line with the finding of increased circulating LPS, short-term alcohol consumption also resulted in a significant increase in G^−^ gut bacteria in IRAK-M−/− mice compared with WT B6 mice, which was reflected as a significant decrease of the G^+^/G^−^ ratio in the gut bacteria (Figure 6D).

## Discussion

The innate immune system senses a wide range of exogenous (pathogens) and endogenous (non-pathogenic insults) “danger” signals through different innate receptors, including the TLRs. TLRs recognize molecular patterns of a variety of microbial products, pathogenic or non-pathogenic, as well as endogenous stress signals, with consequent activation of antigen-presenting cells and produce inflammatory cytokines thus inducing inflammation [31]. All known TLRs, except TLR3, use the MyD88 down-stream pathway, which activates NFkB and leads to cytokine production [32]. IRAK-M is a member of the IL-1 receptor associated kinase (IRAK) family of adaptive molecules. IRAK-M blocks the formation of MyD88/TRAF6 complex and inhibits downstream activation of NF-kB [9], [11] and therefore, IRAK-M is an inhibitor of TLR signaling via MyD88.

Alcoholic liver disease (ALD) constitutes a large proportion of liver disease worldwide. Despite extensive investigation in the past decades, we still do not fully understand the mechanism of the disease and therefore lack effective therapy. The liver is the largest organ in the body and constantly interacts with potentially harmful agents from food and drink. The liver is a highly complex organ that detoxifies those harmful agents. In addition, a large number of immune cells reside in the liver and it has recently been considered to be a lymphoid organ [33], [34]. Moreover, increasing evidence suggests that innate immunity plays an important role in different liver disorders including ALD. Our studies demonstrate the role of IRAK-M, a molecule regulates the activation of MyD88, in hepatic damage induced by acute alcohol consumption. We found that single heavy alcohol consumption triggered liver inflammation and injury in the absence of IRAK-M. The liver injury was evidenced by increased LMNCs infiltration in the liver and elevated alanine aminotransferase in the serum. There was also more CD4^+^ T cell infiltration, shown in Figure 3B, in the liver of IRAK-M deficient mice compared with wild type mice after alcohol consumption. It has been reported that the liver is a reservoir that facilitates CD8 T cell retention [35], [36]. We did not find any obvious alteration of CD8^+^ T cells in the liver; however, among the infiltrated T cells, there was an increase in inflammatory cytokine IFNγ producing CD8^+^ T cells in IRAK-M deficient mice. It is conceivable that these IFNγ producing CD8^+^ T cells contribute to the liver injury induced by alcohol.

CD68^+^ cells in the liver, often considered as Kupffer cells, are resident macrophages and important innate immune cells in the liver. These cells have long been considered as scavenger cells that remove dead cells and potential harmful materials including bacterial products derived from intestine [37], [38]. Kupffer cells also play an important role in pathological conditions including contributing to liver injury by producing pro-inflammatory cytokines in response to different insults to hepatocytes. Despite the increased number of CD68^+^ cells in alcohol induced liver injury in IRAK-M deficient mice, we did not find significant differences in pro-inflammatory cytokine production by CD68^+^ cells. However, it is interesting that the phagocytic function of both conventional macrophages (CD11b^+^CD68^−^) and Kupffer cells (CD11b^−^CD68^+^) from the liver of IRAK-M deficient mice was significantly enhanced.

It is known that the balance of gut bacteria, intestinal permeability, hepatocyte function, and Kupffer cell activation appears to be critical in the maintenance of normal homeostasis of the gut-liver axis. We hypothesized that acute alcohol consumption may affect gut microbiota and cause more bacterial products from the intestine to traffick to liver, which leads to the enhanced phagocytosis by hepatic macrophages and Kuffer cells. To test this hypothesis, we examined the composition of gut microbiota of alcohol treated IRAK-M deficient and sufficient mice. It is interesting that alcohol consumption caused a significant increase in culturable Gram-negative bacteria in both wild type and IRAK-M deficient mice, but the increase of Gram-negative bacteria in the gut of IRAK-M deficient mice was greater. Alcohol consumption also caused a marked increase of gut permeability in IRAK-M deficient mice and this was not observed in wild type mice. Our results suggested that the gut-liver axis was indeed altered by acute alcohol consumption.

In summary, our study provided evidence that IRAK-M plays an important role in alcohol-induced liver injury and IRAK-M negatively regulates the innate and possibly adaptive immune response in the liver reacting to acute insult by alcohol. In the absence of IRAK-M, the hosts developed worse liver injury, altered inflammation, increased gut permeability and altered gut microbiota. We hope that the knowledge gained from this animal study will be useful for human studies.
