# Correction: Role of IRAK-M in Alcohol Induced Liver Injury

**DOI:** 10.1371/annotation/678de7ff-0a7e-4200-a8a2-bd739ff14eef

**Published:** 2014-01-16

**Authors:** Yipeng Wang, Youjia Hu, Chen Chao, Muhammed Yuksel, Isabelle Colle, Richard A. Flavell, Yun Ma, Huiping Yan, Li Wen

Dr. Huiping Yan is co-corresponding author with Dr. Li Wen. Dr. Huiping Yan can be reached at the email address yhp503@vip.sina.com

